# Varying the Interparticle Distances of Gold Nanospheres and Nanorods via Polymer Ligand Chain Lengths

**DOI:** 10.1021/acs.langmuir.5c03961

**Published:** 2026-03-30

**Authors:** Alexandra Leluk, Susanne Seibt, Astrid Rauh, Matthias Karg, Stephan Förster

**Affiliations:** † Jülich Centre for Neutron Science (JCNS-1), Forschungszentrum Jülich, 52428 Jülich, Germany; ‡ Physical Chemistry I, University of Bayreuth, 95440 Bayreuth, Germany; § Physical Chemistry I, 5376 Heinrich Heine University Düsseldorf, 40225 Düsseldorf, Germany; ∥ Institute of Physical Chemistry, RWTH University, 52074 Aachen, Germany

## Abstract

The surface modification and functionalization of nanoparticles with polymer ligands is the focus of current research in order to improve nanoparticle stability and to control nanoparticle assembly. The surface attachment of end-functionalized polymers is a versatile route to structurally well-defined stabilizing polymer shells. Here, we investigate for spherical and cylindrical gold nanoparticles the ligand exchange method to attach end-functionalized polystyrene chains with narrow molecular-weight distribution in order to control the interparticle distance, i.e., nanoparticle surface-to-surface distances, and to stabilize nonspherical particle shapes. For the ligand exchange, we use gold nanoparticles capped with citrate and cetyltrimethylammonium bromide as well as polystyrenes with thiol and multidentate amino end groups. We demonstrate that the interparticle distances for both spherical and cylindrical nanoparticles can be systematically varied via the molecular weight of the polymer ligands. We observe that in both cases, a plateau value of the interparticle distances is reached for large molecular weights. We find that for cylindrical nanoparticles, the polymers preferentially bind to the axial circumference, and to a lesser degree to the cylinder tips, in line with the observed shifts of the longitudinal and transverse plasmon resonances. We show that polymer ligands can be used for the synthesis of spherical seed particles without employing low-molecular-weight capping agents. The results provide further insights into the functionalization of spherical and cylindrical nanoparticles with polymer ligands to provide stability and distance control to tailor nanoparticle assembly.

## Graphical abstract

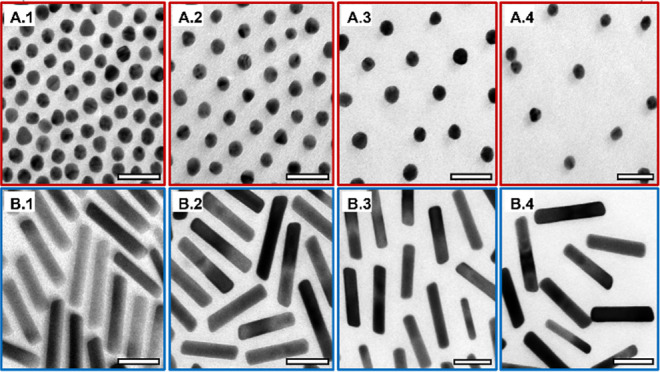

## Introduction

A wide range of highly inspiring research studies about gold nanoparticles (AuNPs) have been published since the description of “beautiful ruby fluid” in 1857.^1^ The broad application potential of colloidal gold arises mainly from its interaction with light, resulting in absorption and elastic light scattering^2,3^ which can be employed for chemical and biochemical sensing,^4,5^ biological imaging,^6^ medical diagnostics, as well as therapeutics.^7,8^ Furthermore, non-biological utilizations such as in the field of solar cells^9–12^ and catalysis^13–16^ are discussed, as well.

The optical properties of gold nanospheres (AuNS) and gold nanorods (AuNR) are determined by collective oscillations of conduction band electrons excited by electromagnetic light waves. These localized surface plasmon resonances (LSPR) appear in the visible to NIR wavelength range depending on shape and size.^17–20^ The optimal use of AuNPs requires narrow size distribution, sufficient colloidal stability, ability for surface functionalization, as well as tunability of the surface-to-surface distance, which here will be referred to as the interparticle distance (*d*_i_). A control of the interparticle distance regulates the nanoparticle volume fraction in nanocomposites and adjusts the plasmon coupling of noble metal nanoparticles, the dipolar coupling for magnetic nanoparticles, and the T2-relaxation time in nanoparticle-based MRI-contrast agents.

Several approaches have been developed to control the surface functionalization and the surface-to-surface distance either by synthesis, postsynthesis surface modification and/or order and alignment of the plasmonic nanoparticles.^21–25^ Furthermore, surface modification and the resulting surface properties play a key role in the assembly of nanoparticles which is relevant, not at last, for the development of functional nanomaterials.^26^ Such modifications can be achieved by commonly used stabilizing agents including sodium citrate,^27,28^ cetyltrimethylammonium bromide (CTAB),^29^ alkanethiols,^30–32^ and mesoporous silica.^33–35^ Further articles describe coating of the NP surface with an organic polymer shell to form nanocomposites.^36–38^ For the attachment of polymer shells, the partial or complete ligand exchange method is increasingly used, for example, to circumvent the cytotoxicity of CTAB^39^ or to enhance the compatibility and arrangement of AuNPs by preferentially binding stabilizing agents to different NP facets,^40,41^ which also offers great versatility in terms of ligand choice and interparticle spacing.

Due to the strong affinity of Au to thiol (SH),^42^ polymers such as poly­(ethylene glycol) (PEG),^43,44^ poly­(*N*-isopropylacrylamide) (PNIPAM),^45,46^ and poly­(styrene) PS^47–49^ have been functionalized with SH-linkage groups for nanoparticle surface attachment. The versatility of SH-mediated polymer attachment has, for example, been demonstrated by the work of Wang et al., who demonstrated that with the assistance of an electric field, the use of a template (anodic aluminum oxide channels) and the ligand exchange of CTAB with SH-terminated PS, alignment of AuNRs in different directions, and an increase of *d*_i_ by increasing polymer molecular weight can be achieved. Accordingly, the edge-to-edge distance was increased from about 7.1 to 17.6 nm, and the tip-to-tip distance increased from about 6.0 to 28.4 nm.^48^ The arrangement of highly anisotropic AuNRs in directional supracolloidal structures is the subject of other recent studies, discussed as a consequence of regioselective binding depending, for example, on the solvent quality^50^ and the oxidation state of the surface ligand,^51^ but also simply on the surface charge of the NRs.^52^ However, these investigations mainly refer to SH-terminated polymers, thus under-representing other functional groups that are promising for both particle positioning and stabilization of the NPs. The latter not only refers to stabilization against aggregation but also with respect to protection of AuNRs against shape transitions to the kinetically and thermodynamically more stable shape of nanospheres. This stabilization is important and seems to be a challenge, for example, with attempts to use an increased silica shell thickness of silica-coated AuNRs and, in general, with ligands with strong Au surface affinity.^22,53^

Following on from this and a previous study by our research group on ligand exchange with functional polymers,^54^ in this article we address the surface modification of spherical as well as rod-shaped AuNPs with narrow disperse polystyrene ligands using multidentate amino surface binding groups as well as SH groups as a reference. As exchangeable low-molecular-weight ligands, we specifically consider negatively charged citrate and positively charged CTAB capping agents that are ligand exchanged with pentaethylenehexamine- (PEHA) and SH-terminated polystyrenes. The PEHA-binding motif is more versatile than the SH-binding motif because it coordinatively binds not only to Au-nanoparticles but also to Ag, CdSe, ZnO, Fe_2_O_3_, and PbS nanoparticles, enabling to prepare well-defined, highly filled nanocomposites.^55^ Transmission electron microscopy (TEM) studies are used to relate the chemical nature and structure of the functional ligands and their interaction with the gold surface to interparticle distance *d*_i_ and the molecular weight of the polymer, and also to the protection of nonspherical NPs against aggregation and shape change to spherical shapes. The corresponding changes in the LSPR properties in the ultraviolet–visible–near-infrared (UV–vis–NIR) spectral region in relation to surface functionalization are also investigated, demonstrating the sensitivity of AuNPs to surface modifications. We attempt to directly use PEHA-functionalized PS instead of conventional low-molecular-weight ligands for the synthesis of AuNR seed particles. This study primarily contributes to the fundamental understanding of how to stabilize and control the interparticle spacing of Au nanoparticle spheres and rods and to gain knowledge about the polymer shell structure for the design of future functional materials.

## Experimental Section

### Chemicals

Hydrogen tetrachloroaurate (III) trihydrate (HAuCl_4_·3H_2_O, Alfa Aesar), hydroquinone (HQ, Sigma-Aldrich), sodium borohydride (NaBH_4_, Sigma-Aldrich), silver nitrate (AgNO_3,_ Sigma-Aldrich), cetyltrimethylammonium bromide (CTAB, Sigma-Aldrich), sodium citrate dehydrate (≥99%, Sigma-Aldrich), pentaethylenehexamine (PEHA, Sigma-Aldrich, technical grade), and 1,1′-carbonyldiimidazole (CDI, Sigma-Aldrich, reagent grade) were used without further purification. Hydroxyl-terminated polystyrene with *M*_n PS–OH 1_ = 11.5 kg·mol^–1^, *M*_n PS–OH 2_ = 22.8 kg·mol^–1^, *M*_n PS–OH 3_ = 33.3 kg·mol^–1^, and *M*_n PS–OH 4_ = 58.3 kg·mol^–1^, thiolated polystyrene (PS–SH) with *M*_n PS–SH_ = 53.9 kg·mol^–1^, and polystyrene-*b*-poly­(acrylic acid) (PS-*b*-PAA) with *M*_n PS_ = 41 kg·mol^–1^ and *M*_n PAA_ = 4.2 kg·mol^–1^ were purchased from Polymer Source, Inc. For the PEHA-functionalization, tetrahydrofuran (THF, Sigma-Aldrich, 250 ppm of BHT as a stabilizer, ≥99.9%), chloroform (CHCl_3_, Sigma-Aldrich, anhydrous, ≥99%, contains amylenes as a stabilizer), as well as deuterated chloroform (CDCl_3_, Roth, 99.8 at. %, stabilized with Ag) were all used as received.

### Spherical Nanoparticle Synthesis

The synthesis of AuNSs was accomplished in accordance to the approach of Turkevich et al.^28^ Prior to synthesis, cleaning of glassware and magnetic stirrers by aqua regia as well as ethanol and finally drying in an oven was ensured. To 500 mL of a HAuCl_4_ solution (5×10^–4^ M), 25 mL of a hot aqueous solution containing 1 wt % of sodium citrate dehydrate was added and stirred under heavy boiling. After a reaction time of 20 min, the resulting dark red solution was cooled while stirring slowly.

### Nanorod Synthesis

AuNRs were synthesized by the silver-assisted seeded growth method according to the protocol of Vigderman and Zubarev^23^ For the preparation of the seed solution, 0.5 mM of HAuCl_4_ were added to 5 mL of an aqueous CTAB solution (0.2 M), stirred vigorously (210 rcf, 10 min), and the mixture turned yellow. Afterward, the reduction of Au was achieved by adding 0.6 mL of ice cold 0.01 M NaBH_4_ and stirring at 210 rcf for approximately 2 min. The solution was left for 1 h. The synthesis of the growth solution was realized by adding 12.5 μL of 0.2 M HAuCl_4_ to 2.5 mL of 0.1 M CTAB. Subsequently, 12.5 μL of 0.1 M AgNO_3_ and 125 μL of 0.4 M HQ were added and the solution was stirred at 35 °C. Finally, 100 μL of seed solution was added on one side to realize AuNRs with aspect ratio (AR) of ∼5.0, and on the other, 175 μL of seed solution was inserted into the growth solution to obtain AuNRs with AR of ∼4.8. The mixtures were centrifuged twice (14.500 rcf, 60 min) and then redispersed in Milli-Q water.

### Modification of OH-Terminated PS

In principle, the PEHA-functionalization method of PS was divided into two parts according to the work of Ehlert et al.,^54^ as described in Scheme 1. The activation step of the hydroxyl group was carried out by the slow addition of a 25-fold excess of CDI dissolved in chloroform. Afterward, the solution was stirred under an inert atmosphere for 24 h. Three extraction steps with demineralized water were carried out, followed by drying under vacuum. PEHA was set to a 25-fold excess and dissolved in CHCl_3_. The activated polymer was again dissolved in the organic solvent and added dropwise to the PEHA solution. The solution was stirred for 24 h under an inert atmosphere and extracted three times with demineralized water. The PEHA-functionalized PS was finally dried under vacuum. ^1^H NMR measurements were performed to monitor the functionalization process.

**Scheme 1:**
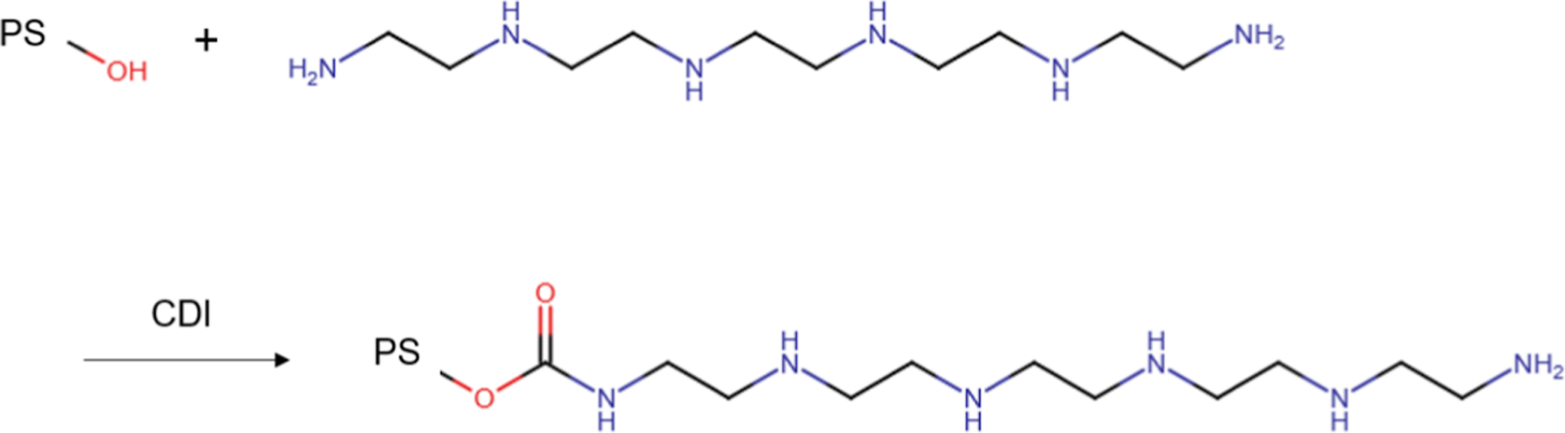
Synthesis of the Multidentate Polystyrene Ligand PS–PEHA (Pentaethylene Hexamine) by Reaction of an OH-terminated Polystyrene with PEHA Using Carbonyldiimadozole (CDI) as an Activation Agent. The OH-Terminated Polystyrene Is Obtained by Using Ethylene Oxide for the Termination Step of the Living Anionic Polymerization of Styrene

### Ligand Exchange and PS-*b*-PAA Coating

Ligand exchanges as well as PS-*b*-PAA coating were performed as reported by Grzelczak et al. with minor modifications.^56^ 1 mL of Au dispersion (0.4 mM citrate-stabilized AuNSs or in the case of AuNRs 4.6 mM CTAB-stabilized rods) was added dropwise to a 10 mL THF solution containing PS-SH or PS–PEHA under sonication, at concentrations corresponding to 5 ligand molecules per nm^2^ nanoparticle surface area. The ultrasound treatment lasted 15 min, and the solution was left for 24 h. The final solution was then centrifuged twice (7100 rcf, 30 min), and the AuNPs were redispersed in THF. The grafting density was determined to be 1.2 ligand chains/nm^2^ by thermogravimetry, as described in the Supporting Information (SI, Section 2).

For coating with PS-*b*-PAA, 450 μL of PS–PEHA@AuNRs dissolved in THF was added to 50 μL of 6 mg/mL PS-*b*-PAA also dissolved in THF. Following this, 250 μL of Mili-Q water was added with magnetic stirring. After a temperature and water content increase up to 75 °C and 50 wt %, respectively, was achieved, the solution was allowed to stand for 30 min and then centrifuged at 4400 rcf for 20 min and redispersed in water.

### PS–PEHA-Stabilized Nanoparticle Synthesis

A modified version of the seed-mediated-growth synthesis described above was used for preparation of both ellipsoidal and spherical nanocrystals.^23^ Thus, for the seed solution, 0.5 mM HAuCl_4_ and 1 mL of 60 mg/mL PS–PEHA were stirred at 210 rcf for 10 min. After the addition of 120 μL of 0.01 M NaBH_4_, stirring was continued for 2 min, and then the solution was left for 1 h. For the growth solution, 2.5 μL of 0.2 M HAuCl_4_, 125 μL of 0.4 M HQ, and 2.5 μL of 0.1 M AgNO_3_ were mixed together with 0.5 mL of 30 mg/mL PS–PEHA. Finally, 100 μL of the seed solution was added and the mixture was centrifuged twice at 14.500 rcf for 30 min.

### Characterization of AuNPs

Investigation of the optical properties of AuNPs was performed using a Cary 5000 spectrophotometer (Agilent Technologies). The measurements of AuNSs were carried out in the wavelength range of 400 to 900 nm and in the case of AuNRs from 400 to 1100 nm. TEM images were taken using a Zeiss CEM902 transmission electron microscope. For TEM investigations, the samples were dispersed in toluene and cast on TEM-grids to form ordered nanocomposite structures via evaporation-induced self-assembly.

### Characterization of the PS–OH Functionalization toward PS–PEHA

A Bruker AC 250 instrument (300 MHz) was used to record the ^1^H NMR spectra of the functionalization process. For this purpose, 10 mg of the polymer was dissolved in 0.7 mL of CDCl_3_.

## Results and Discussion

### Polymer Ligand Exchange and Interparticle Distance Control

In the wet chemical synthesis of AuNPs, chloroauric acid is reduced with a reducing agent in an aqueous or organic phase in the presence of a stabilizer. The stabilizers are used to improve homogeneity and morphological stability and ultimately to prevent aggregation. Weakly adsorbed capping agents such as sodium citrate and CTAB are known to be suitable for ligand exchange as their surface stabilization of AuNPs is based on electrostatic interactions.^32,56^ In addition to these low-molecular-weight capping agents, this study also deals with different molecular-weights *M*_n_ of PEHA-functionalized PS and a selected SH-terminated PS as a reference. For the synthesis of the PS–PEHA ligands, the hydroxyl groups of PS–OH were converted to amino groups for four different molecular weights, which are summarized together with the *M*_n_ of PS–SH in Table 1.

**Table 1:** Number Average *M*_n_, Polydispersity Index (PDI) of PS–OH, as Well as PS–SH Ligands and Their Designations, Respectively

OH-terminated PS functionalized toward PEHA-terminated PS	*M*_*n*__/_ kg·mol^–1^	PDI
PS–OH 1 → PS–PEHA 1	11.6	1.01
PS–OH 2 → PS–PEHA 2	23.2	1.02
PS–OH 3 → PS–PEHA 3	34.8	1.06
PS–OH 4→ PS–PEHA 4	58.7	1.02
PS–SH	54.2	1.06

The thiol and PEHA groups have binding strengths comparable to those of gold. Their binding strengths can be compared within the framework of the hard–soft acid–base (Hard and Soft Acids and Bases (HSAB)) concept. Accordingly, gold is a soft acid that preferentially binds to a soft base. The thiol group is a soft base, therefore binding strongly to gold. Amine groups are somewhat harder bases relative to thiols and therefore bind less strongly to gold. To still have a binding strength comparable to that of thiols, we use the multidentate PEHA amino groups with multiple binding sites. The advantage of the PEHA group is that it binds not only to gold nanoparticles but also to a large range of other metal, oxidic, or sulfidic nanoparticles.

The amine functionalization of initially OH-terminated PS was carried out in two main steps. First, polymer activation was achieved in the presence of an excess of 1,1′-carbonyldiimidazole (CDI) dissolved in chloroform. Second, PEHA dissolved in chloroform was carefully added to the CDI-activated PS solution, and the mixture was stirred overnight and finally dried under vacuum. The accompanying proton NMR spectra of OH-terminated PS and its functionalization toward PEHA-functionalized PS are shown in the Supporting Information (see Figures S1 and S2).

Both citrate-stabilized AuNSs and CTAB-stabilized AuNRs were synthesized to investigate both the geometry dependence and the interchangeability of the initial capping agent via polymeric ligands, PEHA- and SH-capped PS. Pioneering work by Turkevich et al.,^28^ that was later elaborated by Frens,^58^ shows the aqueous reduction of gold salt via sodium citrate, which acts as both stabilizing and reducing agent. Based on the citrate reduction, citrate-capped AuNSs with an average diameter of 17.9 ± 1.5 nm were prepared and used in this study (see Figure 1A.0). Another widely used stabilizer in the synthesis of Au nanocrystals, especially anisotropic NPs, is the positively charged surfactant CTAB, which is adsorbed on the Au surface.^57^ The most commonly applied synthesis method for AuNRs is the so-called silver-assisted seed-mediated growth synthesis,^59^ and in this work, two different sizes were synthesized by alteration of the added seed solution and subsequently analyzed by means of TEM. Thus, AuNRs with an AR of ∼5.0 (average length *L* = 76.7 ± 6.9 nm, width *W* = 15.3 ± 1.8 nm) (see Figure 1B.0) and AR of ∼4.9 (average length *L* = 52.2 ± 6.7 nm, width *W* = 10.7 ± 0.6 nm) (see Figure 1C.0) were obtained. The AuNSs A.0 and the AuNRs B.0 and C.0 were used for the subsequent ligand exchange investigations (see Figure 2 **A.1**–**A.4** and **B.1**–**B.4**). In addition, AuNSs and AuNRs (AR ∼4.9) were treated with PS–SH as a reference to study the comparison of the exchangeability of the two polymeric ligands (Figure 2A.5 and **C.5**).

**Figure 1:**
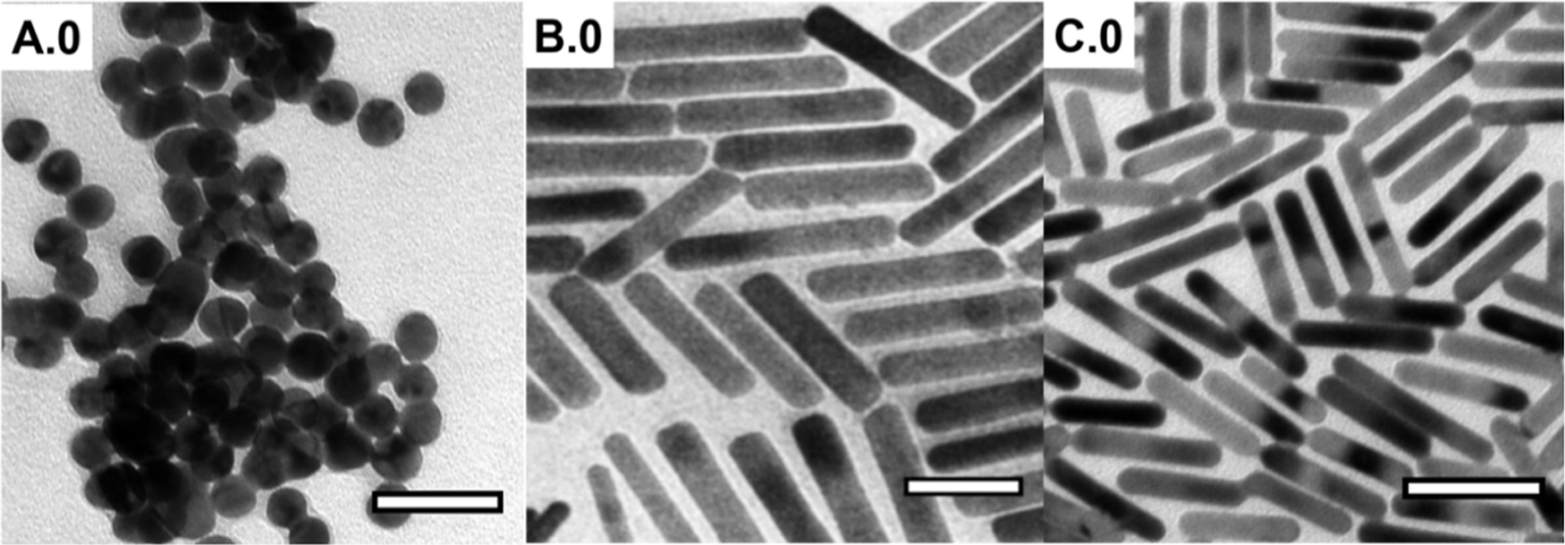
TEM image of citrate-stabilized AuNSs with an average diameter of 17.9 nm ± 1.5 nm is shown in **A.0.** TEM images in **B.0** and **C.0** display CTAB-stabilized AuNRs with AR of ∼5.0 and ∼4.9 prepared with 100 μL and 175 μL seed solution during seed-mediated growth syntheses, respectively. The scale bars correspond to 50 nm.

**Figure 2:**
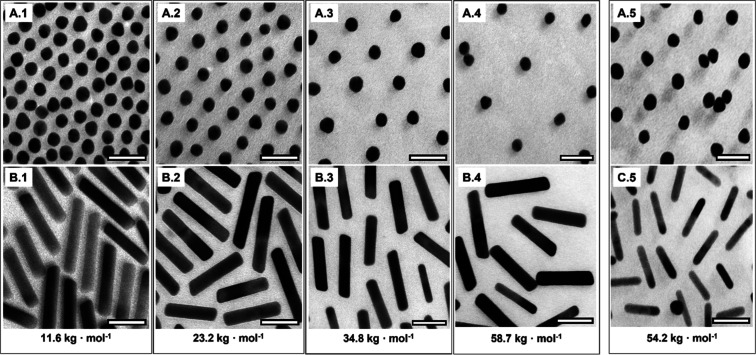
TEM images of **A.1**–**A.4** initially citrate-capped AuNSs and **B.1**–**B.4** initially CTAB-stabilized AuNRs after undergoing ligand exchange with PS–PEHA show increase in *d*_i_ with increasing molecular weight of the polymer from *M*_n PS–PEHA 1_ = 11.6 kg·mol^–1^ to *M*_n PS–PEHA 4_ = 58.7 kg·mol^–1^. Moreover, **A.5** AuNSs and **C.5** AuNRs have been exchanged with PS–SH with *M*_n PS–SH_ = 54.2 kg·mol^–1^ showing an increase in interparticle spacing, as well. The scale bars correspond to 50 nm.

For ligand exchange, as reported by Grzelczak et al.,^56^ dispersions containing citrate-capped AuNSs or CTAB-stabilized AuNRs were added dropwise to organic solutions under sonication to provide ∼5 molecules of PS–PEHA or PS–SH per nm^2^ of Au surface. Representative TEM images of AuNPs exchanged with polymeric ligands are shown in Figure 2.

From Figure 2, it is evident that the distances between the NPs increase as the molecular weight of the polymers increases, regardless of the initial stabilizing agent. Compared to the citrate-stabilized AuNSs in Figure 1A.0, assemblies with well-separated nanoparticles are observed for PS molecular weights *M*_n_ increasing from 11.6, 23.2, 34.8 to 58.7 kg·mol^–1^, in images A.1 to A.4 of Figure 2, respectively. The ligand exchange successfully leads to increased nanoparticle distances for both spherical and rodlike nanoparticles with an AR ∼5 depicted in Figure 2 **B.1–B.4**. For the rodlike nanoparticles, both the side-to-side and the tip-to-tip spacings are increased compared to the low-molecular-weight ligand, while the particle size and AR is maintained. Also, in the case of exchange with PS–SH as a reference, an increased distance between the AuNPs is observed (see Figure 2 **A.5** and **C.5**). Furthermore, a few less separated particles are identified, suggesting limits of functionalization or achievable grafting density at the highest *M*_n_ of the polymer, particularly for SH groups but also for amino groups. For a better overview, at least 50–100 measured surface-to-surface distances from the TEM images were averaged and plotted against the molecular weight of the polymer (see Figure 3b). Specifically, for AuNRs an additional distinction was made between edge-to-edge and tip-to-tip spacing, as depicted in Figure 3a.

**Figure 3:**
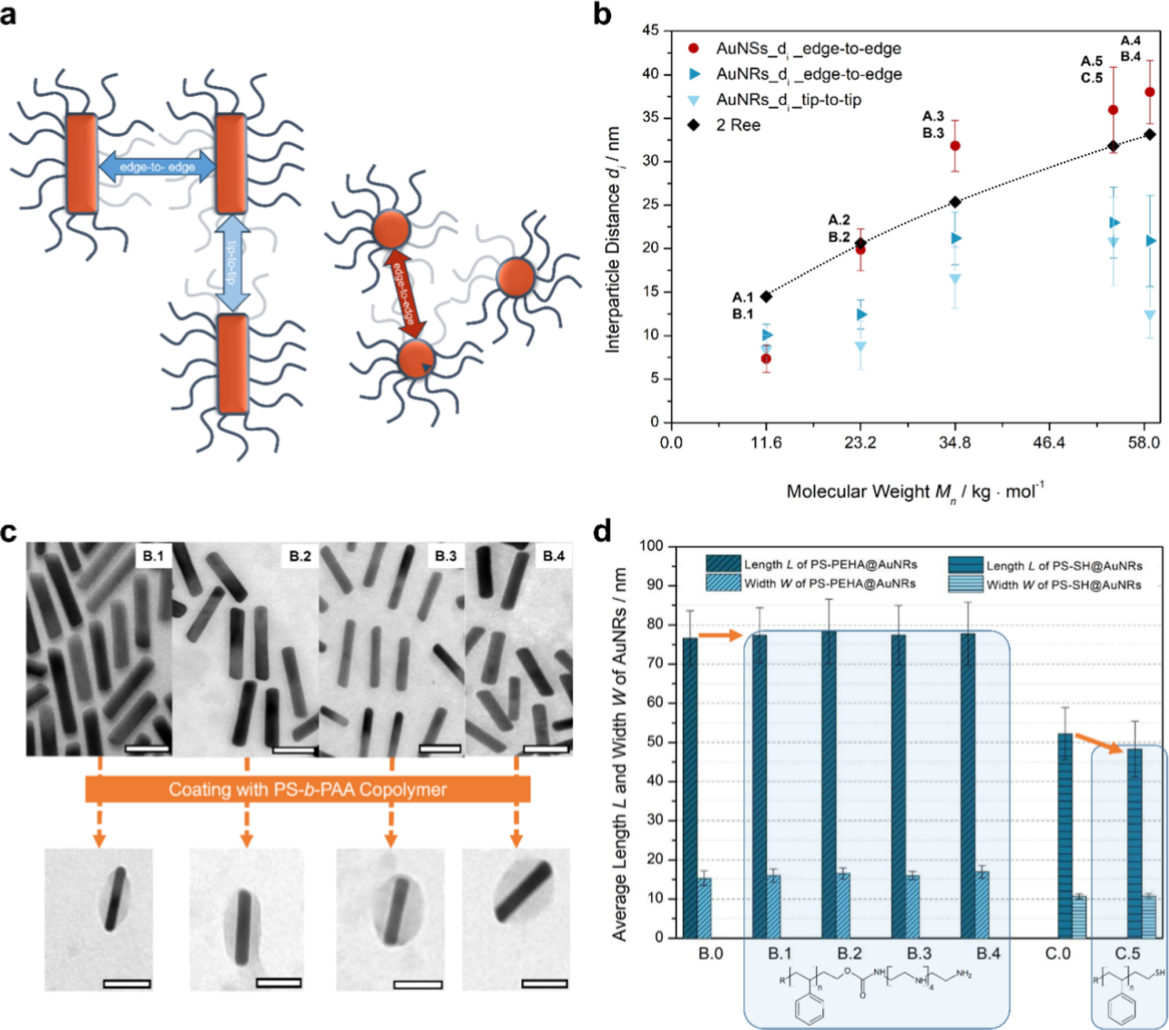
**a**) Schematic representation of the different surface-to-surface distances of AuNRs and AuNSs. (**b**) Plot of d_i_ for AuNSs and AuNRs as a function of *M*_n_ of PEHA- and SH-terminated PS. (**c**) Visualization of PS–PEHA@AuNRs which were also coated with a PS-*b*-PAA copolymer to contrast the polymer shell via TEM images (scale bar = 50 nm). (**d**) Bar graphs of the lengths and widths of AuNRs after exchange with PS–PEHA showing no decrease in either parameter, but a slight decrease in nanorod length when exchanged with PS–SH. The end-to-end distance values *R*_ee_, which are connected by the solid line in (**b**), correspond to calculated values in dilute solutions, with *R*_ee_^2^ = 6 *R*_g_^2^ and *R*_g_ = 0.25·*M*_n_^0.51^ following ref 59

As shown in Figure 3b, an analysis of the nanoparticle edge-to-edge distance *d*_i_ of the spherical AuNPs as a function of the polymer molecular weight clearly reveals a systematic increase in *d*_i_ that exhibits a value of 7.3 ± 1.6 nm for *M*_n PS–PEHA 1_ (A1) and continues to increase from 19.9 ± 2.4 nm (A2) to 31.8 ± 2.9 nm (A3), eventually reaching a plateau at 38.2 ± 3.7 nm (A4) as the highest value in the case of *M*_n PS–PEHA 4_, comparable to the value of 35.9 ± 4.9 nm for the PS–SH system (A5). The observed distances are in a similar range reported for poly­(methyl acrylate)-grafted silica nanoparticles^60^ and for polystyrene-grafted silica nanoparticles^61^ but leveling off at a lower interparticle distance.

The influence of geometry can be investigated in the case of ligand exchange with cylindrical AuNPs. For the lowest *M*_n PS–PEHA 1_ (B1), the distances of 10.1 nm ± 1.2 nm (edge-to-edge) and 8.4 nm ± 1.6 nm (tip-to-tip) are in a comparable range as in the case of AuNSs, indicating that both citrate and CTAB are well exchanged by the PS–PEHA ligands. For cylindrical nanoparticles, the edge-to-edge distances increase comparatively less compared to spherical nanoparticles, with increasing *M*_n_ reaching a plateau at 54.2 kg·mol^–1^ (C5) and decreasing at 58.7 kg·mol^–1^ (B4), especially in the case of the tip-to-tip down to 12.5 ± 2.8 nm.

In general, we observe in Figure 3b that *d*_i_(AuNS) > *d*_i_(AuNR,edge-to-edge) > *d*_i_(AuNR,tip-to-tip) for the same ligand molecular weights. As indicated by the dotted line in Figure 3b, the interparticle distances are on the order of two times the end-to-end distance *R*_ee_ of the polymer chains in dilute solution. *R*_ee_ is calculated from the radius of gyration *R*_g_ as *R*_ee_^2^ = 6*R*_g_^2^, with the relation *R*_g_ = 0.25·*M*_n_^0.51^ in units of Å.^62^

The difference in the spatial distance between the spherical and cylindrical nanocrystals could also be attributed to the polymer shell density. In this regard, due to the surface curvature, spherical surfaces have a radial *r* ^2^-increase of the area per chain, which reduces to an *r* ^1^-relation for cylindrical surfaces and a *r* ^0^-relation for the planar surfaces of the cylinder tips, thus reducing the surface accessibility of the polymer chains. Similarly, as the grafting of PS–SH chains onto initial citrate-stabilized spheres from Yockell–Lelièvre’s work shows, more polymer chains can reach the gold surface with a higher surface curvature as they are less sterically hindered in the process.^63^

For a more detailed visualization of the shape and thickness of the PS–PEHA polymer shells, the corresponding AuNRs were additionally coated with polystyrene-*b*-poly­(acrylic acid) (PS-*b*-PAA) (*M*_n PS_ = 41 kg·mol^–1^ and *M*_n PAA_ = 4.2 kg·mol^–1^). For this, the PS–PEHA-coated Au nanorods were dissolved in THF, together with the PS-*b*-PAA block copolymer, and subsequently transferred into water according to the procedure in ref 56 Upon transfer into water, the hydrophobic PS-shell collapses and forms an inner dense shell, while the hydrophilic water-swollen outer PAA-shell stabilizes the nanoparticles against aggregation. In the TEM images, the dense PS-shell shows an increased contrast and enables visualization of the distribution of polystyrene chains on the side and on the tip of the nanorods, as shown in Figure 3c. The thickness of the polymer shell is largest at the side of the PS–PEHA ligand-coated nanorods, with a maximum thickness in the center of the nanocrystals. In the case of AuNRs whose CTAB ligand was exchanged with PS–PEHA 3 (Figure 3c, B.3), a slight increase in the polymer shell at the tips of the AuNRs is also observed.

Overall, it can be summarized that as *M*_n_ increases, the polymer shell thickness first increases nearly linearly with increasing *M*_n_, reaching a plateau value at the highest molecular weights. The initial linear increase *d*_i_ ∼ *M*_n_^1^ indicates an extended chain configuration in dense polymer shells, as for lower density polymer shells, a weaker *d*_i_ ∼ *M*_n_^1/2^-dependence would be expected. The findings are also consistent with the study by Wang et al.^48^ where it was demonstrated that an increase in edge-to-edge and tip-to-tip distances could be achieved with an increase in PS–SH molecular weight. In our investigation, the edge-to-edge distance reached a value of 22.8 nm at the highest selected molecular weight ∼50 kg·mol^–1^, compared to a maximum edge-to-edge distance of 17.6 nm reported in ref 43. The high affinity between SH groups and Au surface^42^ and their preferential binding to the tips of AuNRs, e.g., in two-dimensional assembly or self-assembly processes,^40,64^ are increasingly discussed as reasons for these spatial variations. Although the preferential attachment of the SH group to the tips of the AuNRs cannot be deduced from a single reference measurement, it can be seen that the PS–PEHA ligand stabilizes the NR especially in the nanorod center rather than at the nanorod tips, and we assume that this preferential attachment in the middle region of the AuNRs, even in the case of higher *M*_n_, is the reason for the good protection against shrinkage and shape loss of the nanorods, even though the solutions were centrifuged several times.

The protection of polymer ligands against shape loss, i.e., an unwanted nanoparticle rod-to-sphere transition, was investigated in more detail. Thus, both the lengths and widths of the AuNRs were also determined after ligand exchange which are summarized in Figure 3d. After surface modification with PS–PEHA, neither the length nor the width of initially CTAB-capped AuNRs (**B.0**) are changed. After surface modification stabilization of the shorter AuNRs (**C.0**) with PS–SH, there is rather a slight decrease in average length from *L* = 52.2 ± 6.7 nm down to *L* = 48.3 ± 7.1 nm, while the width remains the same. Therefore, in addition to strongly binding SH, also moderately binding PEHA ligands according to Pearson’s HSAB concept^66^ can successfully be used to stabilize AuNPs, especially also those with anisotropic shapes. Graphene liquid cell electron microscopy observations show similar results that sufficient stabilization at the center of AuNRs and/or active diffusion along the Au surface appear to be successful strategies for maintaining anisotropy.^67^

### Surface Plasmon Resonances of Spherical and Rodlike Nanoparticles

The effects of ligand exchange with PS are also reflected in the optical properties of the nanoparticles, as shown in Figure 4. The UV–vis–NIR absorbance spectra were normalized for better comparison of the changes with respect to the surface plasmon resonance positions. UV–vis–NIR spectroscopy is an effective method to monitor local surface binding and density differences caused by the ligand exchange. The effect of nanoparticle shape on the plasmon resonances is well understood. AuNPs of spherical shape show a single resonance peak (referred to as transverse plasmon) related to a dipolar LSPR up to a certain size where additional modes such as quadrupolar modes start to become relevant.^68^ For anisotropic AuNPs as the studied AuNRs, an additional higher wavelength mode (longitudinal) is observed due to the oscillation of valence electrons along the long axis of the rods. The sensitivity of AuNPs to particle size and shape, dielectric environment, particle spacing, and particle arrangement is reflected in the absorption spectra.^17–20^ Referring to Figure 4a, the LSPR peak for citrate-stabilized AuNSs is located at 522 nm in line with the expected value^69^ and shifts slightly to 527 nm with PS–SH as polymer ligand dissolved in THF. A slight broadening of the peak is observed after PS–SH exchange, which may be attributed to the formation of small aggregates of the nanoparticles, as observed in Figure 2. The broadening of the peak is also seen upon substitution with PS–PEHA 3 and PS–PEHA 4, together with the appearance of a characteristic Rayleigh λ^–4^-scattering as an increasing background, which we attribute to a tendency of multiplet formation, as visible in the TEM images in Figure 2 for the two samples. Peak broadening can also be due to inhomogeneous surface functionalization of surface patchiness, as discussed in refs 70 and 71 After ligand exchange with PS–PEHA 1 and PS–PEHA 2, no broadening of the LSPR peak is observed. A slight but measurable red-shift to 535 nm occurs for all PS–PEHA ligands, indicating dense surface attachment of the polymer ligand chains and the effect of the change in refractive index.^49^

**Figure 4:**
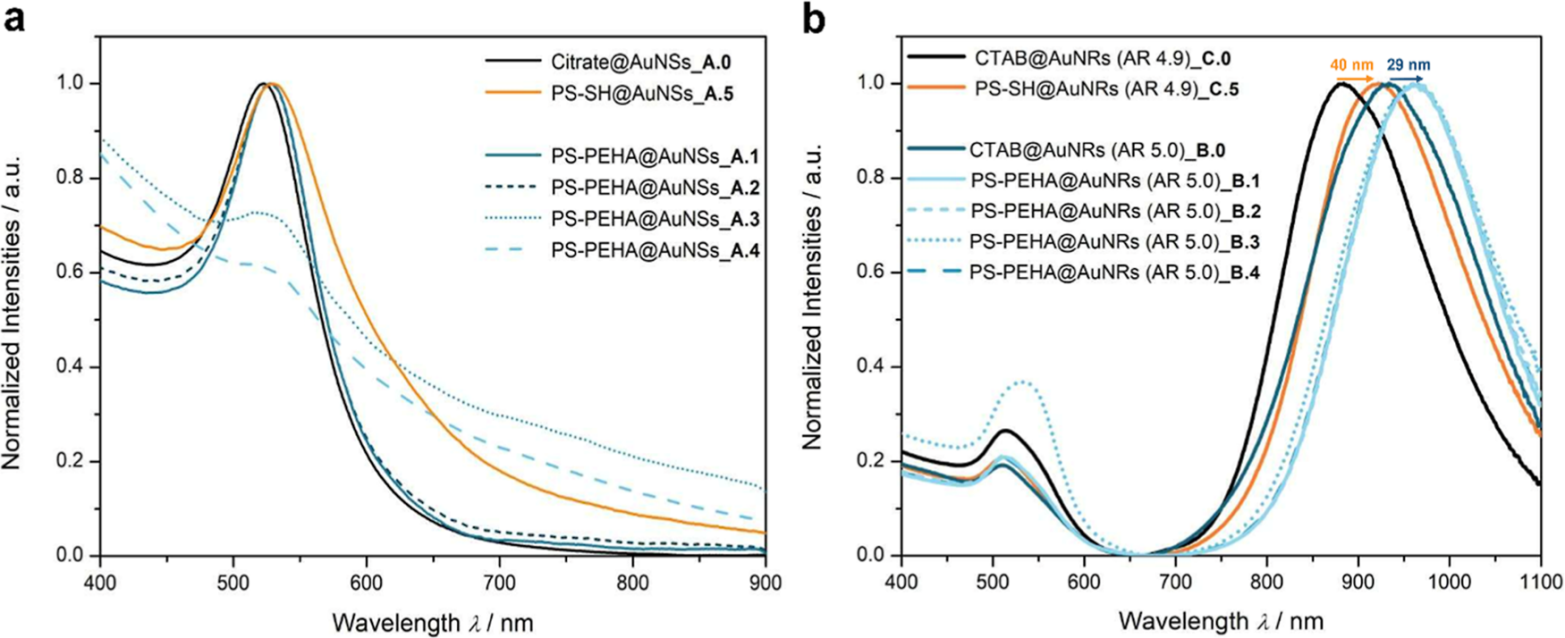
Normalized UV–vis–NIR absorbance spectra of dilute solutions of (a) spherical and (b) rodlike NPs before and after ligand exchanges with both PEHA-and PS-capped PS, showing no additional peaks compared to nonexchanged NPs, which would indicate a loss of shape upon exchange with higher *M*_n_. Especially in case of AuNRs, not only red-shift of the longitudinal LSPR peak but also changes in the optical properties of PS-capped AuNSs are obvious due to the change in the dielectric environment.

Furthermore, the effect of polymer layer attachment is clearly observable for both sizes of AuNRs and is most evident in the red-shift of the longitudinal plasmon resonance (Figure 4b). The longitudinal LSPR peak of shorter NRs (AR ∼4.9) is shifted from 882 to 922 nm, and for AuNRs of the AR ∼5.0, a red-shift from 933 to 962 nm is detectable. Therefore, the dense binding of the polymer ligands to the surface along the cylinder axis is clearly visible in both cases, in agreement with the TEM images in Figure 3b. There are no additional peaks, indicating byproducts and polydispersity. Only very small differences can be seen in the transverse plasmon resonance. Only after the exchange with PS–PEHA 3, a change in shape as well as a red-shift to 536 nm can be observed, which may indicate the functionalization of the ends of the NRs, as shown in Figure 3 **b B.3**. When it is exchanged with PS–SH and the highest *M*_n_ of PS–PEHA, this shape change is not observed.

### Nanoparticle Synthesis in the Presence of Polymeric Ligands as Stabilizers

Since the successful ligand exchange with PEHA-terminated PS indicated good surface binding of the PEHA-group toward Au, the seed-mediated synthesis of AuNPs in the presence of PS–PEHA, without low-molecular-weight capping agents was investigated. The syntheses of polymer-attached spherical AuNPs with SH-terminated polymers^72^ as well as homopolymers^73^ and block copolymers^74^ with amino groups in so-called one-pot syntheses procedures have already been described, and the advantages of direct polymer attachment such as efficiency and stability without the need for further postmodification are convincing. Here, we attempted to realize spherical seed particles that could be employed for the synthesis of AuNRs that are directly stabilized with polymeric ligands without the use of capping agents such as CTAB or citrate. Therefore, the synthesis protocol of Vigderman and Zubarev^23^ for AuNRs was slightly modified. First, a seed solution was prepared by mixing a solution containing hydrogen tetrachloroaurate hydrate with PS–PEHA 3 as a stabilizer. Then, the reducing agent NaBH_4_ was added. The growth solution was then prepared by similarly stabilizing the gold ion solution with the polymer ligand and adding silver ions in combination with a stronger reducing agent, hydroquinone, to promote anisotropic growth. Finally, 100 μL of seed solution was added to the growth solution, and the AuNPs were allowed to grow.

For comparison between direct and postmodified polymer attachment, the nanoparticles obtained by direct polymer ligand binding on the one hand and the established ligand exchange with polymeric ligands on the other hand are shown in the TEM images in Figures 2A.3 and 5.

**Figure 5:**
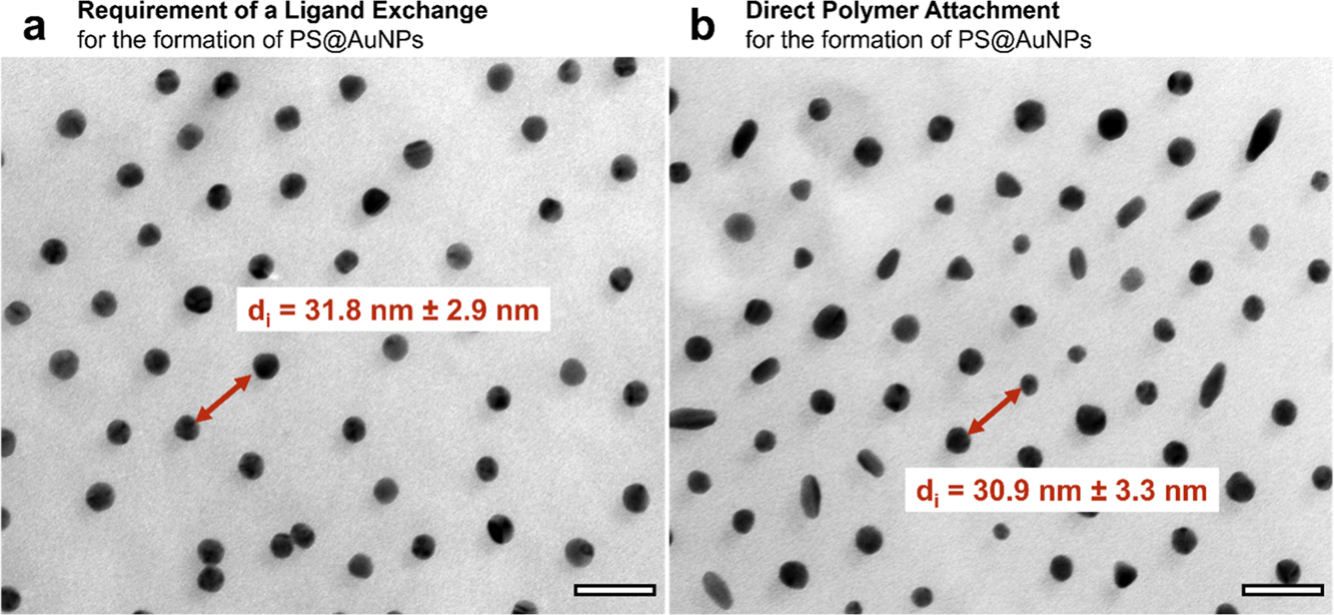
TEM images of (a) PS–PEHA@AuNPs, initially stabilized with the citrate ligand, then exchanged for the polymer ligand PS–PEHA 3 and (b) PS–PEHA@AuNPs, directly stabilized without ligand exchange with the same PS–PEHA 3 ligand. The highlighted average edge-to-edge distance between two spherical AuNPs for both approaches shows comparable values. The scale bars correspond to 50 nm.

For the case of directly polymer ligand-stabilized NPs, it is evident that mostly spherical but also some anisotropic or ellipsoidal nanocrystals are formed. The measurement of 50 spherical particles results in a mean diameter of 17.2 ± 3.0 nm and thus a comparable mean diameter of 17.9 ± 1.5 nm for the citrate-capped AuNSs (Figure 1A.0). This shows, in principle, that for spherical nanoparticles, the citrate ligand could be replaced by the PS–PEHA polymeric ligand to achieve similar nanoparticle diameters. The measurement of 50 ellipsoidal AuNPs results in an average width *W =* 13.8 ± 1.2 nm and an average length *L* = 31.6 ± 3.1 nm. Some of the anisotropic nanoparticles appear to be in the early symmetry breaking stage, others are already in the stage of elongated growth. Obviously, the polymeric ligand represents a steric barrier for the diffusion of the Au precursors present in the growth solution such that the transition into anisotropic shape growth is retarded. This presently hinders the synthesis of high AR nanoparticles. This is similar to the arresting effect of sulfide^70^ or thiolated^71^ ligands. An advantage of the direct attachment of the functionalized polymer is the direct setting of the desired *d*_i_. Here, the average edge-to-edge distance between two spherical AuNPs is *d*_i_ = 30.9 ± 3.3 nm (see Figure 5b) and is therefore comparable to that of the ligand-exchanged AuNSs of *d*_i_ = 31.8 ± 2.9 nm (see Figure 5a). Since subsequent modification is time-consuming, cost-intensive, and there is also a risk that the shape stability of anisotropic AuNPs will be lost, e.g., by removing the stabilizing layer during cleaning processes, direct capping with polymeric ligands may enable the rapid and simple synthesis of nanoparticles with the desired surface functionality.

## Conclusion

In summary, this work addresses the attachment of polymeric ligand shells onto spherical and cylindrical nanoparticle surfaces by exchange with SH- and PEHA-terminated PS ligands from previously citrate-stabilized AuNSs and CTAB-stabilized AuNRs. By variation of the molecular weight of the PS, the particle spacings can be systematically adjusted for spherical and cylindrical nanoparticles, reaching plateau values for the highest molecular weights in both cases. Analysis of the LSPR by absorption spectroscopy revealed that the binding of the polymer ligands to the surface of the spherical nanoparticles and to the lateral surface of the cylindrical nanoparticles leads to significant red-shifts of the respective plasmon resonances caused by the change in the local refractive index environment. Together with TEM investigations, this indicates that for cylindrical nanoparticles, the polymer ligands preferentially bind to the axial surface and to a lesser degree to the cylinder tip surface. The preferred binding of the PS–PEHA ligands to the lateral surface stabilizes cylindrical nanoparticles against shape changes to spherical nanoparticles. This highlighted difference in polymer ligand attachment might also contribute to the explanation of current relevant issues, such as the discrepancy in the observation of the diffusion coefficients for translational and rotational diffusion of thermoresponsive PNIPAM ligands attached on AuNRs.^65^ It was further shown that the use of PEHA-functionalized PS in both the seed and growth solutions in the seed-mediated growth method enabled the synthesis of directly polymer-attached gold nanoparticles, producing diameters and *d*_i_ similar to citrate-capped AuNSs. This may be useful for direct synthesis methods of polymer-stabilized AuNPs or more generally for the preparation of 2D and 3D assembly materials.

## Supplementary Material



## Notes

Depiction of proton nuclear magnetic resonance (^1^H NMR) measurements dealing with the functionalization procedure of OH toward PEHA-terminated polystyrene (PDF)
