# Supplementary material for: Varying the Interparticle Distances of Gold Nanospheres and Nanorods via Polymer Ligand Chain Lengths
Source: Langmuir. 2026 Mar 30;42(14):9637–46. doi: 10.1021/acs.langmuir.5c03961 (PMC13085792; doi:10.1021/acs.langmuir.5c03961)
Supplement: Supplementary file 1 [file la5c03961_si_001.pdf]

Supporting Information

Varying the Interparticle Distances of Gold Nanospheres and Nanorods *via* Polymer Ligand Chain Lengths

Alexandra Leluk[1], *Susanne Seibt*[2], *Astrid Rauh*[3], *Matthias Karg*[3], *Stephan Förster*[1,4,*]

[1] Jülich Centre for Neutron Science (JCNS-1), Forschungszentrum Jülich, 52428 Jülich, Germany
[2] Physical Chemistry I, University of Bayreuth, 95440 Bayreuth, Germany
[3] Physical Chemistry I, Heinrich Heine University Düsseldorf, 40225 Düsseldorf, Germany
[4] Institute of Physical Chemistry, RWTH University, 52074 Aachen, Germany

[*]Address correspondence to: s.foerster@fz-juelich.de

1. NMR-spectra of PS-PEHA

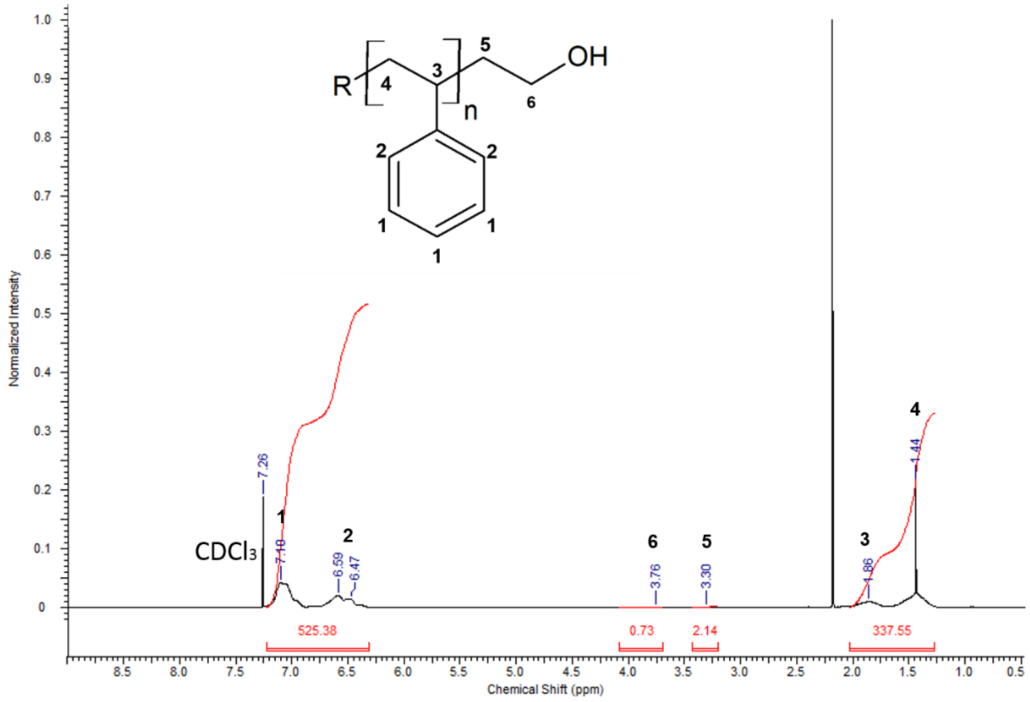

Figure S1. 300 Hz proton NMR spectrum of hydroxyl-terminated PS with $M_n = 11.5\ kg \cdot mol^{-1}$.

Besides of the well-defined peak at 7.3 ppm that is provoked by $CDCl_3$, characteristic peaks for the protons of the PS benzene ring at 7.1 ppm (1), 6.6 ppm and at 6.5 ppm (2) are apparent. Furthermore, aliphatic protons of the backbone at 1.86 ppm and 1.44 ppm are visible (3 and 4). Signals caused by protons next to the hydroxyl end group appear weaker at 3.8 ppm (6) and 3.3 ppm (5) and protons next to the OH-end group undergo a shift to the downfield.[72] The signal at 2.2 ppm is caused by residual acetone from cleaning of the NMR tubes.[73]

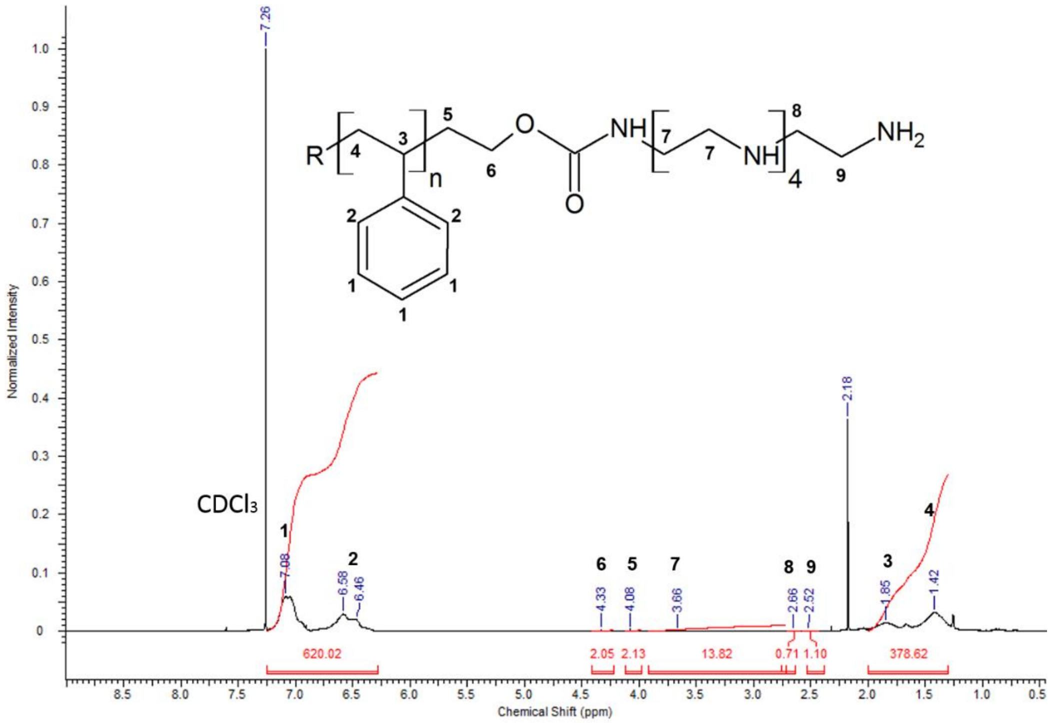

Figure S2. 300 Hz proton NMR spectrum of PEHA-functionalized PS.

After PEHA-functionalization of OH-PS, the proton NMR spectrum shows a broad peak at 3.7 ppm (7). This signal can be attributed to protons in the PEHA end group and the peaks at 2.66 ppm (8) and 2.52 ppm (9) are caused by the methylene groups next to it. The remaining protons of the methylene groups caused by CDI-activation are still shifted to 4.33 ppm (6) and 4.08 ppm (5).

2. **Thermogravimetry (TGA) and determination of the grafting density**

a) b)

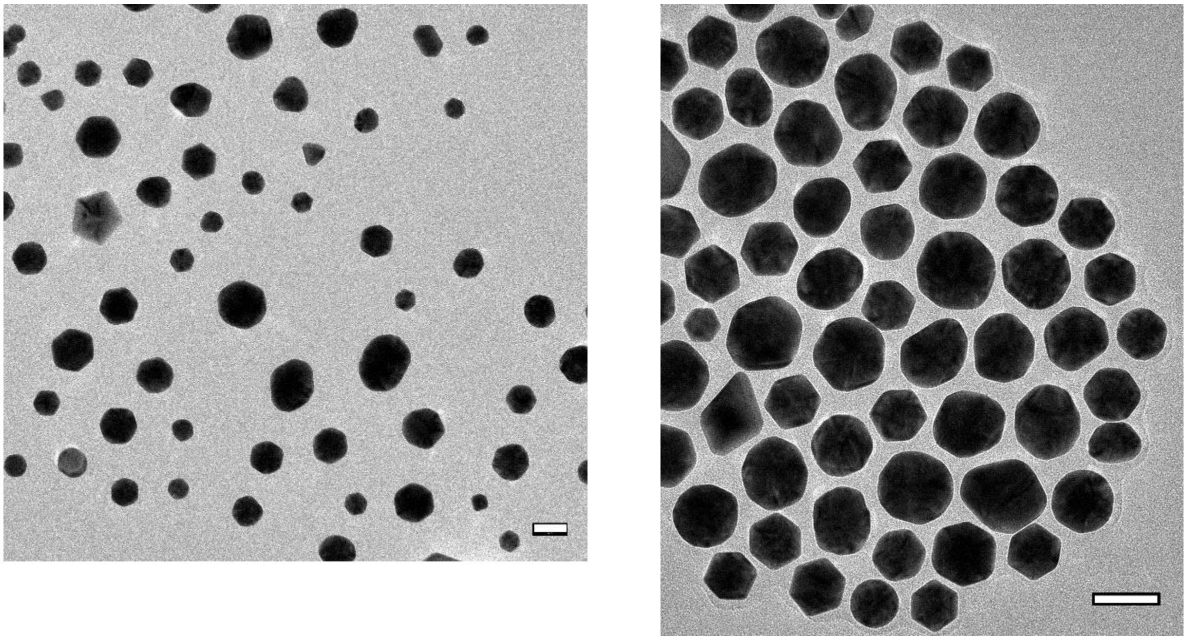

Figure S3. TEM-images of polystyrene-coated gold nanoparticles Au50PS53k (a) and Au50PS26k (b)

a) b)

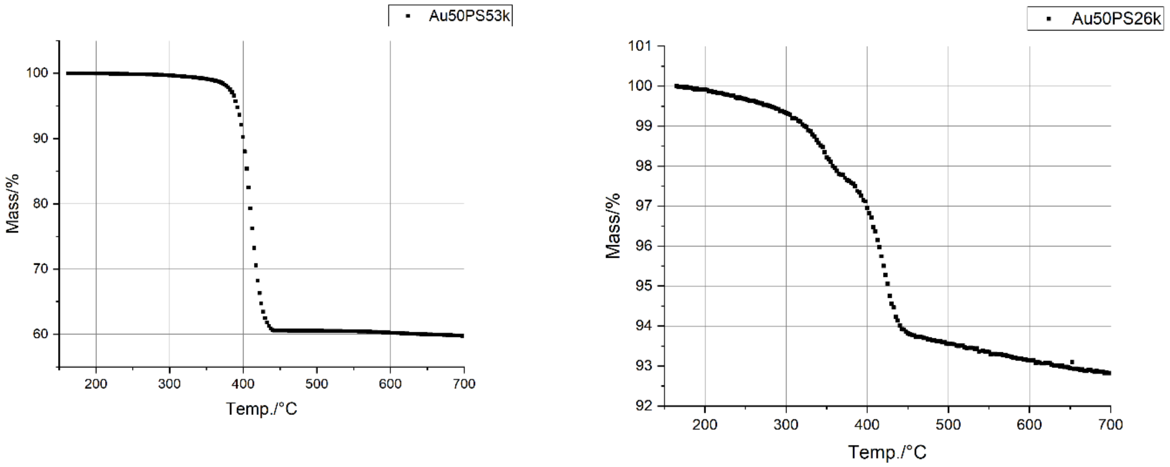

Figure S4. TGA-curves of polystyrene-coated gold nanoparticles Au50PS53k (a) and Au50PS26k (b).

Fig. S3 shows the TEM-images of the two newly synthesized Au nanoparticle samples Au50PS53k and Au50PS26k. After ligand exchange, sample Au50PS26k was washed several times to remove PS-PEHA ligands from the nanoparticle surface to reduce the grafting density. Fig. S4 shows the TGA curves of the

two samples. The determined relative nanoparticle:polymer weight fractions are ϕ=60:40 for Au50PS53k and ϕ=93:7 for Au50PS26k.

Using a bulk density for gold of ρ=19.3 g/cm^3, a nanoparticle radius of $R_{Au} = 25$ nm and the molecular weight M of the polymer, we can calculate the surface grafting density from

$$\rho_s = \frac{R_{Au} N_A}{3\phi M}$$

We therefore obtain a grafting density of 1.2 nm^{-2} for Au50PS53k and of 0.3 nm^{-2} for Au50PS26k. This indicates, that ligand washing indeed leads to a significant reduction of the polymer grafting density.
